# High Sensitivity Humidity Detection Based on Functional GO/MWCNTs Hybrid Nano-Materials Coated Titled Fiber Bragg Grating

**DOI:** 10.3390/nano11051134

**Published:** 2021-04-27

**Authors:** Fang Wang, Bowen Wang, Xuhui Zhang, Mengdi Lu, Yang Zhang, Changsen Sun, Wei Peng

**Affiliations:** 1 School of Optoelectronic Engineering and Instrument Science, Dalian University of Technology, Dalian 116024, China; wangfang1020@mail.dlut.edu.cn (F.W.); suncs@dlut.edu.cn (C.S.); 2 School of Physics, Dalian University of Technology, Dalian 116024, China; bwwang@mail.dlut.edu.cn (B.W.); zhangxuhui@mail.dlut.edu.cn (X.Z.); mdlu@dlut.edu.cn (M.L.); wpeng@dlut.edu.cn (W.P.)

**Keywords:** fiber Bragg grating, graphene oxide, humidity detection, multi-walled carbon nanotubes

## Abstract

A high performance humidity sensor using tilted fiber Bragg grating (TFBG) and functional graphene oxide (GO)/multi-walled carbon nanotubes (MWCNTs) hybrid nano-materials was proposed. The humidity-sensitive material with three-dimensional (3D) structure was synthesized by the MWCNTs and GOs. Comparing with traditional two dimensional (2D) GOs film, water molecules could be absorbed effectively due to the larger ripples and more holes in GO/MWCNTs layers. The water molecule will fill the entire space in the 3D structure instead of air, which further enhances the absorption efficiency of the hybrid nanomaterial. TFBG as a compact and robust surrounding complex dielectric constant sensing platform was utilized. The mode coupling coefficient or the amplitude of TFBG cladding mode will vary sharply with the imaginary part of permittivity of the hybrid nanomaterial, realizing the high performance RH sensing. In the experiments, we successfully demonstrated that this 3D structural nanomaterial composed by the MWCNTs and GOs has significant advantages for expanding the range of humidity detection (range from 30% to 90%) and enhancing the detection sensitivity (0.377 dB/% RH is twice more than humidity sensor with 2D GO film). The TFBG-based RH sensor also exhibits good repeatability and stability. Our proposed humidity sensor has potential application in environmental and healthy monitoring fields.

## 1. Introduction

Humidity monitoring has taken an important role in numerous areas such as corrosion protection, precision electronics manufacturing, food processing, and other industries [1,2,3]. Traditional humidity sensors are mainly based on electric, gravimetric, and thermal conductive technology including capacitive type, resistive type, and photoelectric type humidity sensors [4,5,6,7,8]. Among those humidity sensors, electric humidity sensors with high sensitivity and accuracy are widely produced in the current market. However, these sensors are not suitable for humidity detection in harsh environment (such as flammable and strong electromagnetic situation). Nowadays, fiber-optic humidity sensors attract extensive attention due to their unique advantages of stability, compact size, electromagnetic immunity, fast response, and remote sensing ability [9,10,11]. Commonly, fiber-optic humidity sensors consist of two important parts: the light coupling elements and the humidity-sensitive materials. In the humidity measurement, the light coupling elements guide the light coupling into the humidity-sensitive material and discriminate the change of environmental humidity levels. Optical fiber interferometric structures like Mach-Zehnder interferometer (MZI) and Fabry-Perot interferometer (FPI) have been proposed for humidity measurement [12,13,14]. Although these structures are featured with high sensitivity, the temperature cross-talk and complex welding process still need to be addressed. In order to reduce the negative effects of these methods, fiber Bragg grating (FBG)-based humidity sensors have been demonstrated [15,16]. For general FBG, the grating plane is perpendicular to the fiber axis and it is difficult to couple the light from fiber core to surface with a robust structure unless its physical fiber structure is broken. The tilted fiber Bragg grating (TFBG) as a special FBG could easily excite a lot of high order cladding modes with the titled grating plane. This unique feature enables the TFBG to directly sense the complex permittivity of the surrounding medium. In recent years, it has been widely applied for multiple-parameter sensing including bending, refractive index (RI), and bio-chemical measurements [17,18,19,20]. In addition, the temperature compensation capability makes it more attractable in practical applications. These features make TFBG as an ideal coupling and sensing element for relative humidity (RH) sensing.

On the other hand, it is crucial to select a suitable humidity-sensitive material for RH measurement. The humidity-sensitive materials with a lot of hydrophilic functional groups, good swelling degree, and large specific surface area are attractive for measuring the RH. The widely used humidity sensing materials are porous ceramics, semiconducting materials, polymers (polyvinyl alcohol, polyethylene oxide, gelatin, etc.), and novel carbon materials (graphene, carbon nanotubes, and graphene oxide films) [21,22,23,24,25]. W. Wong et al. proposed a polyvinyl alcohol coated photonic crystal optical fiber as a humidity sensor. The sensitivity of this sensor was 0.60 nm/% RH, and it showed good repeatability and stability [26]. H. F. Liu et al. used the SiO_2_ nanoparticles coated S-Taper fiber as an RH sensor achieving the sensitivity of 0.441 dB/% RH and a range of 83.8% RH to 95.2% RH [27]. Y. Luo et al. has demonstrated an RH humidity detection method based on WS_2_ coated side polished fiber (SPF) with a good linear correlation coefficient of 99.39% and a sensitivity of 0.1213 dB/% RH [28]. However, the poor penetrability and small specific surface area of these materials prevent the water molecules absorption and hence limit the improvement of sensitivity. In recent years, carbon materials have become one of the most widely used multi-functional nanomaterials because of their promising properties. Graphene oxide (GO) as a derivative of graphene is stable in aqueous solution and polar solvent. It has a large number of oxygen-containing groups on surface and edge that can permeate and absorb water molecules, making GO a good candidate for humidity-sensitive material [29,30]. For example, C. Y. Shen et al. reported a humidity sensor with the maximum sensitivity of 0.129 dB/% RH based on GO coated TFBG [31]. K. Prabuddha et al. used the LPFG as the light coupling element and GO as the humidity-sensitive material design a humidity sensor. In the RH range from 60% RH to 95% RH, this proposed sensor has shown a sensitivity of 0.15 dB/% RH (R^2^ = 0.980) [32]. The S taper fiber sensor based on GO film (maximum intensity sensitivity –0.361 dB/% RH, maximum wavelength sensitivity 365 pm/% RH) was proposed for measuring RH by Y. Zhao et al. [33]. In those humidity sensors, the RI of the GO will be changed by the interaction of GO and water molecules resulting in the amplitude/wavelength variations of resonance peaks with environmental humidity levels. However, the effective sensing surface area of these novel nano-materials, which is directly determined by the sensing ability of the proposed RH sensor, is still limited by the flat surface of 2D configuration. Moreover, the unavoidable stacking of these kinds of GO sheets will also significantly reduce the effective absorption surface.

Herein, we proposed a TFBG-based humidity sensor with GO and evenly dispersed multi-walled carbon nanotubes (MWCNTs) hybrid humidity-sensitive nanomaterial. TFBG could couple most of the lights in the core to the cladding and make the lights directly interact with the humidity-sensitive material. The humidity sensing film was synthesized by GO/MWCNTs hybrid nanomaterial with three-dimensional (3D) structure. The MWCNTs play an essential role in supporting and expanding the space between the GO sheets. Via the supporting MWCNTs and GO films, the configuration makes water molecule fill the entire space instead of air which might strengthen the absorption effect of the molecule onto the hybrid nanomaterial. We will creatively combine TFBG with GO/MWCNTs hybrid nanomaterial to measure the relative humidity, and experimentally study the perturbation mechanism of the material’s dielectric constant. The proposed sensor with good performance will show good potential applications for health and environmental monitoring in many fields.

## 2. Principle and Experimental System

For a TFBG, the resonance wavelength *λ_r_* meeting the phase matching condition could be expressed as:

(1) $$\lambda_{r}=(N_{eff}^{core}(\lambda_{r})+N_{eff}^{r}(\lambda_{r}))\Lambda /\cos(\theta )$$

where *θ* is the tilt angle of the TFBG, Λ is the period of TFBG. $N_{eff}^{core}(\lambda_{r})$ and $N_{eff}^{r}(\lambda_{r})$ are the core mode effective RI and cladding mode effective RI at the wavelength *λ_r_*, respectively [17].

The intensity *R* of the TFBG cladding modes are related to the length *L* of the TFBG and the coupling coefficient *κ* between the exciting cladding mode and core mode. It can be expressed as follows:

(2) $$R=\tanh^{2}(\kappa L)$$

The coupling coefficient is calculated as follows:

(3) $$\kappa =C\iint {\vec{E}}_{core}^{*}{\vec{E}}_{r}\Delta n(x,y)dxdy$$

where *C* is a constant depended on the electric field distribution of core mode *E_core_* and cladding mode *E_r_*. Δn(x,y)=Δncos((4π/Λ)(zcos(θ)+ysin(θ))) is the function that describes the RI perturbation. For the proposed humidity sensor, GO/MWCNTs film could absorb water molecules and the effective RI in both the real part and imaginary part of GO or GO/MWCNTs film will be changed, resulting in the strength of the grating resonances varying.

The conductivity σ of GO could be calculated through Equation (4):

(4) $$\sigma =j\frac{e^{2}k_{B}T}{\pi h^{2}(\omega -j2\pi )}[\frac{\mu_{c}}{k_{B}T}+2\ln(e^{-\mu_{c}/k_{B}T}+1)]+j\frac{e^{2}}{4\pi h}\ln[\frac{2\left|\mu_{c}\right|-(\omega +j2\tau )h}{2\left|\mu_{c}\right|+(\omega +j2\tau )h}]$$

where *e* is the unit charge, *μ_c_* is the change of the chemical potential, *h* is Planck’s constant, and *k_B_* is Boltzmann’s constant. *T* and *τ* are the environment temperature and vibration frequency [29].

GO or GO/MWCNTs film will absorb more and more water molecules with the increasing of RH, and the GO layer is filled by the absorbed water molecules. The density of the GO increases with the increasing humidity, which leads to the decrease of the conductivity (σ∝Re(nGO)×Im(nGO)) [30]. For tightly overlapped stacks of single GO in approximately 2D plane, the real and imaginary part of the effective RI of the thin GO film might both decrease with the conductivity as reported in recent articles [17,31]. While for the hybrid nanostructure case with the 3D configuration that is supported by the added MWCNTs, more water molecules will be absorbed in two ways: by the enriched surface/ripples/holes of both GO and MWCNTs, and by filling the entire space instead of air. On the one hand, the enhanced absorption in the first way which is similar to that in single GO film will of course further reduce the conductivity of the composite material resulting in the decrease of both the real part and the imaginary part of RI. On the other hand, different from the situation with the tightly overlapped GO stacks, the large numbers of water molecules (RI ~1.33) will fill into the air occupied space (RI, ~1) of the 3D structure, which is supported by the MWCNTs. It will consequently increase the real part of RI of the composite film, which might compensate the decrease of real part of RI resulted from the conductivity reduction to some extent. Thus, the most significant effect of adding MWCNTs is the great decrease of the conductivity hence the imaginary part change of the RI of the composite film. It is certain that the decrease of both real part and imaginary part of the surface material film RI would contribute to the increment of imaginary part of effective RI of high order cladding modes, i.e., the effective modal loss coefficient which will consequently weaken the transmission depth of the resonances (compressed) [18]. For the sensing responses, effective improvements of the RH sensitivity and the dynamic sensing range over than that of the case with single GO film are to be expected according to the analysis above.

**Figure 1:**
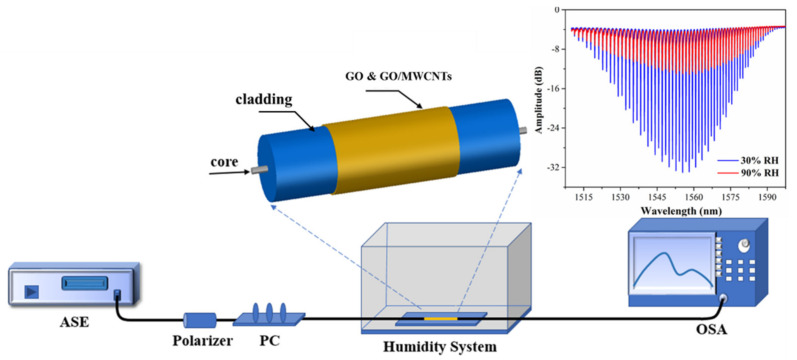
Experimental device for fiber-optic humidity sensor based on GO/MWCNTs coated TFBG.

The experimental system is shown in Figure 1. The broadband light source is used as input light (ASE, wavelength range 1510–1600 nm). A fiber-optic sensor probe is placed into a controllable thermostat and humidity box (purchased fromHoyatek Inc. Shenzhen, China), and the humidity range is programmed to be 30–90% RH. A spectrum analyzer is used as the optical receiver (OSA, YKAQ6370, Yokogawa Inc. Japan). TFBG has two polarization modes (P mode and S mode) which have a strong influence on the transmission spectrum. Additionally, the two polarization modes of TFBG are determined by the polarization state of the input core mode [17]. Here, we use a polarization controller (PC, Hoyatek Inc. Shenzhen, China) to control the input light to remain in the P-polarized state for a better spectrum. By a phase mask technique, TFBG is fabricated using a hydrogen-loaded single-mode fiber. In the fabrication process, a 248 nm UV excimer laser pulse (6 mJ and 150 Hz) is used to inscribe a 1.5 cm long TFBG by scanning technique. In order to remove residual hydrogen and maintain TFBG temperature stability, the fabricated TFBG is annealed at a high temperature stove.

**Figure 2:**
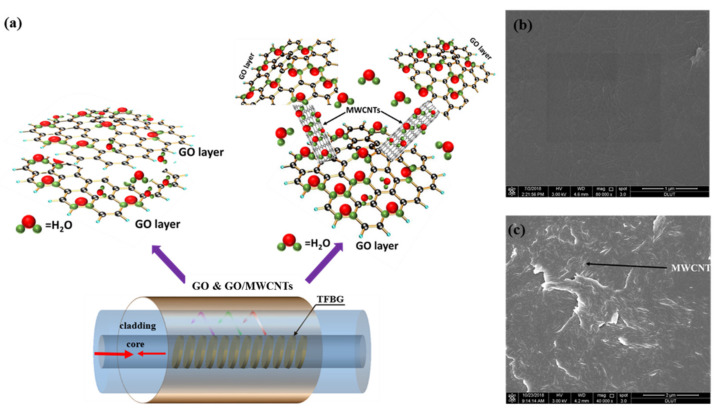
The GO or GO/MWCNTs hybrid nanomaterial sensing film. (**a**) Schematic diagrams ‘of the humidity sensor based on GO or GO/MWCNTs; (**b**,**c**) SEM diagrams of surface of TFBG coated with GO or GO/MWCNTs.

The physical precipitation method is selected to coat the GO or GO/MWCNTs on the fiber surface. First, the TFBG sensor is immersed into hydrochloric acid solution (20 min) and then flushed with plenty of deionized water and ethanol. Second, the sensor is immersed in GO aqueous solution or GO/MWCNTs solution to obtain GO or GO/MWCNTs film. At the same time, the sensor with GO aqueous solution or GO/MWCNTs solution is dried in a drying oven for one hour. Finally, to remove unsecured GO or GO/MWCNTs film from the TFBG’s surface, the sensor is washed with ethanol or deionized water and blown dry with nitrogen. The GO/MWCNTs aqueous solution was prepared by simple sonication. CNTs could adhere to the flat layers of GO through strong π-π stacking interaction [34,35]. Therefore, without any additional organic solvents added, GO could be regarded as dispersants for CNTs in aqueous solution. Besides, the additional MWCNTs could play a supporting role and significantly enlarge the distance between the GO sheets to have better properties in humidity detection, as seen in Figure 2a. Here, the ratio of 2.5:1 between GO and MWCNTs is selected for excellent performance [36]. As shown in Figure 2a, TFBG has unique structure that the grating plane titled to the fiber axis. This characteristic makes lights easily couple from the core to the cladding and interact with GO or GO/MWCNTs film on the surface of the fiber. GO is hydrophilic and can be easily modified as requires because it contains a lot of oxygen-containing functional groups. Figure 2b,c present the SEM images after TFBG coating with GO and GO/MWCNTs. There are some wrinkles on the TFBG surface after coating GO as shown in Figure 2b. It can be clearly seen in Figure 2c that the GO could be regarded as dispersants for MWCNTs in aqueous solution and the MWCNTs support the distance between the GO layers.

## 3. Experimental Results and Discussion

**Figure 3:**
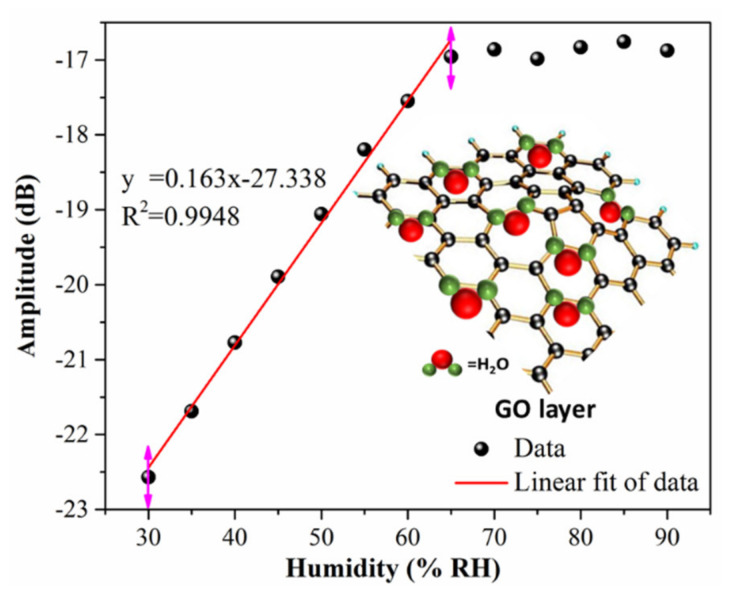
Relative humidity sensitivity of TFBG coated with GO film.

In this paper, the humidity response of GO coating TFBG was first performed. Figure 3 indicated that the relative humidity sensitivity of TFBG coated with GO film. It exhibited good linearity under the RH changing from 30% to 65%, and the RH sensitivity was 0.163 dB/% RH. Although the GO-coated TFBG sensor could be used for humidity detection in the environment, the limits of sensitivity and humidity detection range still need to be resolved. Carbon nanotube (CNT) is a one-dimensional quantum material with many abnormally mechanical, electrical, and chemical properties. In recent years, with the in-depth research on carbon nanotubes, their broad application prospects have been shown. However, the CNTs solution is difficult to dissolve and usually generates large aggregates due to the intertubular Van der Waals interactions and high chemical inertness, which greatly restrict their sensing application. Using GO as a surfactant to disperse CNTs has been reported [33]. The combination of MWCNTs and GO offers larger surface area and makes water molecules easily absorbed. It is benefiting to strengthen the sensitivity and enlarge the range of the humidity detection.

**Figure 4:**
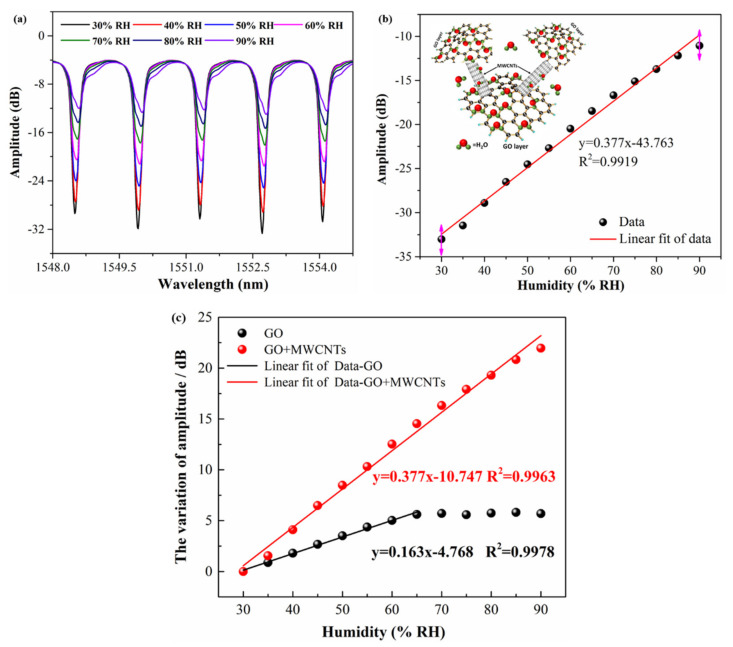
Humidity sensing performances. (**a**) Spectral response of TFBG coated with GO/MWCNTs film to different relative humidity. (**b**) Relative humidity sensitivity of TFBG coated with GO/MWCNTs film. (**c**) Relative humidity sensitivity comparison between TFBG coated with GO and GO/MWCNTs.

Figure 4a shows the resonance spectrogram of the TFBG sensor with GO/MWCNTs film under different humidity. It can be clearly observed that the amplitudes of the TFBG resonance peaks decrease obviously with increasing of the relative humidity. That is to say, the water molecules dramatically act on the GO/MWCNTs 3D composite material to influence the imaginary part of cladding modes effective refractive index hence the modes loss coefficient, which mainly change the amplitude of cladding mode. From the spectrum evolution, we also observed that the wavelength of cladding mode resonances barely changed with RH which is a little different from the wavelength responses with that in the case with GO film [31]. This phenomenon could be explained by the two different water molecules absorption ways in 3D supported nanomaterial film as MWCNTs added. The decrease of the real part of the film RI might be partly compensated by the increase of real part of RI induced by spatial absorption of water molecules by the supported film. In another word, the significant improvement of the resonance peak amplitude response is dominated by the imaginary part of the effective RI of the high order cladding mode for TFBG under GO/MWCNTs modification. As shown in Figure 4b, the amplitude of 1552.61 nm cladding mode resonance peak compresses as much as 23 dB when the surrounding relative humidity changes from 30% to 90%. The linear fitting result exhibits a sensitivity of 0.377 dB/% RH with R^2^ of 99.19%. Comparing with the humidity sensor based on TFBG and GO film, the sensitivity under GO/MWCNTs film coating is twice more than that under GO film condition and the sensor detection range of RH is effectively expanded by coating with GO/MWCNTs film as shown in Figure 4c (The variation of saturation strength under GO/MWCNTs film coating of ~23 dB is four times larger over than that of GO film coating of ~5 dB).

**Figure 5:**
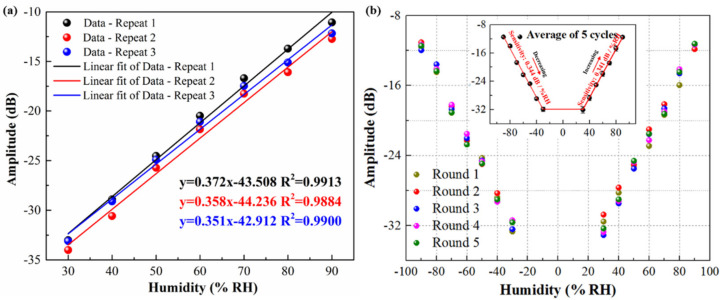
Repeat tests with different relative humidity. (**a**) Three repeated experimental data under increasing the relative humidity (**b**) Five cycle tests under increasing and decreasing the RH.

To evaluate the repeatability and reusability of the proposed sensor, three times repeat tests with different relative humidity were performed. It can be seen in Figure 5a that the three repeated experimental data under an increasing process of the relative humidity exhibit high consistency. The sensitivities are 0.371 dB/%RH, 0.358 dB/% RH, and 0.351 dB/% RH with the linear correlation coefficients of 0.9919, 0.9884, and 0.9900, respectively. The standard deviation of sensitivity under three repetitive experiments can be calculated to 0.01015. Figure 5b shows five cycle tests under increasing and decreasing the RH. With the increasing of RH, GO/MWCNTs hybrid film will absorb more and more water molecules, and the amplitudes of TFBG cladding mode resonances are compressed. In five cycle experiments between the minimum and the maximum, the sensor also exhibits excellent consistency. It can be concluded that this proposed humidity sensor has good potential in practical application.

**Figure 6:**
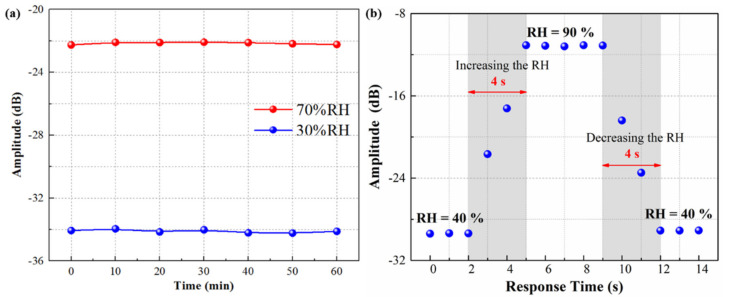
Stability and the response time of the TFBG humidity sensor coated functional GO/MWCNTs hybrid nano-material. (**a**) Stability test in a constant temperature and humidity chamber (25 °C temperature, 30% and 70% RH, 60 min) (**b**) Dynamic response times (the RH changes from 90% to 40%).

Finally, the stability and the response time of the proposed fiber optic humidity sensor were also investigated. First, the TFBG coated with GO/MWCNTs film humidity sensor is placed in a constant temperature and humidity chamber (25 °C temperature, 30% and 70% RH, 60 min). Figure 6a shows the small fluctuation of the amplitude of 1552.61 nm resonance peak (maximum 0.7% float). This small fluctuation may result from the instability of the ASE. Moreover, the dynamic response time is a key parameter that needs to be discussed. It is shown in Figure 6b that our sensor achieves a short response time of 4 s as the relative humidity changes from 90% to 40% (include increasing and decreasing process). Benefiting from the temperature compensation ability of the TFBG-Bragg mode, this proposed sensor with good stability and fast response characteristic exhibits potential value for environmental humidity detection. In Table 1, comparing with other fiber-optic based RH sensors, our proposed RH sensor based on TFBG and 3D structured nanomaterial film exhibits obvious advantages of high sensitivity, simple structure, high stability, fast response, and large measurement range. In order to meet the requirements in the various field applications, such as storage of chemicals and gases, manufacture of transformers and batteries, safe operation of gas insulated switchgear in power industry and coal mine, etc., our future works will focus on simplification of the sensing system and the development of novel humidity sensor under low humidity levels.

## 4. Conclusions

In this paper, a GO/MWCNTs-based TFBG humidity sensor with high sensitivity and large dynamic range has been demonstrated. In this humidity measurement, the light coupling element is the TFBG which easily excites cladding modes by coupling the core light to the cladding and interacts with the humidity-sensitive material on the fiber surface. The addition of MWCNTs enlarges the distance between the GO sheets and promotes the absorption of water molecules by the enriched surface/ripples/holes of both GO and MWCNTs. Thus, the composite of GO and MWCNTs has obvious advantages for expanding the humidity detection range and improving the detection sensitivity. The experimental results show that the sensitivity of RH detection sensor is 0.377 dB/% RH, which is twice more than that with GO film and the range of humidity detection is 30–90%. Moreover, the proposed humidity sensor possesses excellent repeatability, stability, and fast response characteristics. Therefore, this humidity sensor has potential applications for health and environmental humidity monitoring.

## Figures and Tables

**Table 1:** Comparisons of fiber RH sensors.

Sensor Type	Material	Sensitivity	Range	Ref
TFBG	PEDOT:PSS	0.015 dB/% RH	20–80% RH	[37]
TFBG	PAHP4	0.00272 dB/% RH	20–80% RH	[37]
TFBG	GO	0.129 dB/% RH	10–80% RH	[31]
S fiber taper	GO	0.361 dB/% RH	43–95% RH	[33]
MZI	GO	0.263 dB/% RH	35–85% RH	[14]
LPFG	GO	0.15 dB/% RH	60–95% RH	[32]
TFBG	GO/MWCNT	0.377 dB/% RH	30–90% RH	This work

## Data Availability

Data are available in the main text.

## References

[1] Zhang L., Gu F., Lou J., Yin X., Tong L. (2008). Fast detection of humidity with a subwavelength-diameter fiber taper coated with gelatin film. Opt. Express.

[2] Yeo T.L., Sun T., Grattan K.T.V. (2008). Fibre-optic sensor technologies for humidity and moisture measurement. Sens. Actuators Phys..

[3] Enjin A., Zaharieva E.E., Frank D.D., Mansourian S., Suh G.S., Gallio M., Stensmyr M.C. (2016). Humidity sensing in drosophila. Curr. Biol..

[4] Fanget S., Hentz S., Puget P., Arcamone J., Matheron M., Colinet E., Andreucci P., Duraffourg L., Myers E., Roukes M.L. (2011). Gas sensors based on gravimetric detection—A review. Sens. Actuators Biol. Chem..

[5] Duraia E.S.M., Beall G.W. (2015). Humidity sensing properties of reduced humic acid. Sens. Actuators Biol. Chem..

[6] Wang Q., Pan Y.Z., Huang S.S., Ren S.T., Li P., Li J.J. (2010). Resistive and capacitive response of nitrogen-doped TiO_2_ nanotubes film humidity sensor. Nanotechnology.

[7] Rivadeneyra A., Fernandez-Salmeron J., Agudo-Acemel M., Lopez-Villanueva J.A., Capitan-Vallvey L.F., Palma A.J. (2016). Printed electrodes structures as capacitive humidity sensors: A comparison. Sens. Actuators A Phys..

[8] Kolpakov S., Gordon N., Mou C., Zhou K. (2014). Toward a new generation of photonic humidity sensors. Sensors.

[9] Sikarwar S., Yadav B.C. (2015). Opto-electronic humidity sensor: A review. Sens. Actuators A Phys..

[10] Chen G.Y., Lancaster D.G., Monro T.M. (2017). Optical microfiber technology for current, temperature, acceleration, acoustic, humidity and ultraviolet light sensing. Sensors.

[11] Alberto N., Tavares C., Domingues M.F., Correia S.F.H., Marques C., Antunes P., Pinto J.L., Ferreira R.A.S., André P.S. (2016). Relative humidity sensing using micro-cavities produced by the catastrophic fuse effect. Opt. Quantum Electron..

[12] Fan X., Wang Q., Zhou M., Liu F., Shen H., Wei Z., Wang F., Tan C., Meng H. (2020). Humidity sensor based on a graphene oxide-coated few-mode fiber Mach-Zehnder interferometer. Opt. Express.

[13] Chen L.H., Li T., Chan C.C., Menon R., Balamurali P., Shaillender M., Neu B., Ang X.M., Zu P., Wong W.C. (2012). Chitosan based fiber-optic Fabry–Perot humidity sensor. Sens. Actuators B Chem..

[14] Deng S., Meng H., Wang X., Fan X., Wang Q., Zhou M., Guo X., Wei Z., Wang F., Tan C. (2019). Graphene oxide-film-coated splitting ratio adjustable Mach-Zehnder interferometer for relative humidity sensing. Opt. Express.

[15] Gu B., Yin M., Zhang A.P., Qian J., He S. (2011). Optical fiber relative humidity sensor based on FBG incorporated thin-core fiber modal interferometer. Opt. Express.

[16] Berruti G., Consales M., Giordano M., Sansone L., Petagna P., Buontempo S., Breglio G., Cusano A. (2013). Radiation hard humidity sensors for high energy physics applications using polyimide-coated fiber Bragg gratings sensors. Sens. Actuators B Chem..

[17] Albert J., Shao L.Y., Caucheteur C. (2013). Tilted fiber Bragg grating sensors. Laser Photonics Rev..

[18] Alam M.Z. (2012). In Situ Characterization of Single-Wall Carbon Nanotube Thin Film Growth Using the Polarization Properties of Tilted Fibre Bragg Gratings. Ph.D. Thesis.

[19] Zhang Y., Wang F., Qian S.Y., Liu Z.X., Wang Q., Gu Y.Y., Wu Z.L., Jing Z.G., Sun C.S., Peng W. (2017). A Novel Fiber Optic Surface Plasmon Resonance Biosensors with Special Boronic Acid Derivative to Detect Glycoprotein. Sensors.

[20] Zhang Y., Wang F., Liu Z.G., Duan Z.H., Cui W.L., Han J., Gu Y., Wu Z.L., Jing Z.G., Sun C.S. (2017). Fiber-optic anemometer based on single-walled carbon nanotube coated tilted fiber Bragg grating. Opt. Express.

[21] Bjorkqvist M., Salonen J., Paski J., Laine E. (2000). Characterization of thermally carbonized porous silicon humidity sensor. Sens. Actuators A Phys..

[22] Wu S., Wang G., Xue Z., Ge F., Zhang G., Lu H., Qiu L. (2017). Organic field-effec transistors with macroporous semiconductor films as high-performance humidity sensors. ACS Appl. Mater. Interf..

[23] Fratoddi I., Bearzotti A., Venditti I., Cametti C., Russo M.V. (2016). Chemical role of nanostructured polymers on the improvement of electrical response-based relative humidity sensors. Sens. Actuators B Chem..

[24] Nakajima T., Nakamura T., Tsuchiya T. (2016). Flexible humidity sensors composed of graphite-like carbon micro-pinecone arrays. RSC Adv..

[25] Zhang D., Wang K., Tong J., Xia B. (2016). Layer-by-layer nanoassembly fabrication and humidity sensing behaviors of multi-walled carbon nanotubes/polyelectrolyte hybrid film. J. Nanosci. Nanotechnol..

[26] Wong W.C., Chan C.C., Chen L.H., Li T., Lee K.X., Leong K.C. (2012). Polyvinyl alcohol coated photonic crystal optical fiber sensor for humidity measurement. Sens. Actuators Biol. Chem..

[27] Liu H.F., Miao Y.P., Liu B., Lin W., Zhang H., Song B.B., Huang M.D., Lin L. (2015). Relative Humidity Sensor Based on S-Taper Fiber Coated with SiO_2_ Nanoparticles. IEEE Sens. J..

[28] Luo Y., Chen C., Xia K., Peng S., Guan H., Tang J., Lu H., Yu J., Zhang J., Xiao Y. (2016). Tungsten disulfide WS_2_-based all-fiber-optic humidity sensor. Opt. Express.

[29] Li Z.Q., Henriksen E.A., Jiang Z., Hao Z., Martin M.C., Kim P., Stormer H.L., Basov D.N. (2008). Dirac charge dynamics in graphene by infrared spectroscopy. Nat. Phys..

[30] Nair R.R., Wu H.A., Jayaram P.N., Grigorieva I.V., Geim A.K. (2012). Unimpeded permeation of water through helium-leak-tight graphene-based membranes. Science.

[31] Wang Y.Q., Shen C.Y., Lou W.M., Shentu F.Y., Zhong C., Dong X.Y., Tong L.M. (2016). Fiber optic relative humidity sensor based on the tilted fiber Bragg grating coated with graphene oxide. Appl. Phys. Lett..

[32] Prabuddha K.S., Dissanayake W., Wu W.P., Nguyen H., Sun T., Grattan K.T.V. (2018). Graphene oxide–coated long period grating-based fiber optic sensor for relative humidity and external refractive index. J. Lightwave Technol..

[33] Zhao Y., Li A.W., Ming X.Y., Zhu Y.Q., Sun X.C., Li P., Yu Y.S. (2019). Relative humidity sensor of S fiber taper based on graphene oxide film. Opt. Commun..

[34] Zhang C., Ren L., Wang X., Liu T. (2010). Graphene oxide-assisted dispersion of pristine multiwalled carbon nanotubes in aqueous media. J. Phys. Chem. C.

[35] Qiu L., Yang X., Gou X., Yang W., Ma Z. (2010). Dispersing carbon nanotubes with graphene oxide in water and synergistic effects between graphene derivatives. Chem. Eur. J..

[36] Li X.Y., Chen X.D., Chen X.P., Ding X., Zhao X. (2018). High-sensitive humidity sensor based on graphene oxide with evenly dispersed multiwalled carbon nanotubes. Mater. Chem. Phys..

[37] Wen H.Y., Huang W.Y., Huang T.S., Hsu Y.C., Chiang C.C. (2020). Comparison of the sensing mechanisms and capabilities of three functional materials surface-modifed TFBG sensors. AIP Adv..

